# Gingerol and Chitosan-Based Coating of Thermoformed Orthodontic Aligners: Characterization, Assessment of Anti-Microbial Activity, and Scratch Resistance: An In Vitro Study

**DOI:** 10.7759/cureus.42933

**Published:** 2023-08-04

**Authors:** Nazleen Valerie Vas, Ravindra Kumar Jain, Sathish Kumar Ramachandran

**Affiliations:** 1 Department of Orthodontics, Saveetha Dental College and Hospitals, Chennai, IND; 2 Department of Biomaterials, Saveetha Dental College and Hospitals, Chennai, IND

**Keywords:** gingerol, chitosan, removable orthodontic appliance, biopolymer, clear aligners

## Abstract

Aim

To prepare and characterize a 6-gingerol-incorporated chitosan biopolymer for coating on thermoformed aligners and evaluate its scratch resistance and antimicrobial activity.

Material and methods

In this in vitro study, 6-gingerol extract was prepared, incorporated with chitosan biopolymer into a coating solution and characterized using nuclear magnetic resonance imaging spectroscopy (NMR). Twenty thermoformed aligner samples were exposed to UV radiation for surface activation, then coated with a crosslinking agent. These were divided into four groups of five. The control group consisted of samples dip-coated in a chitosan solution for 15 minutes. The three test groups consisted of samples dip coated in a gingerol-chitosan coating solution, with each group representing the following time periods of dip coating: five, 10, and 15 minutes. The crosslinking of the coating with the aligner material was confirmed by a Fourier transform infrared spectroscopy (FTIR) test. A scratch test was carried out to evaluate the wear resistance of the coating, and the antibacterial properties of the coating were tested using a Disc Diffusion test.

Results

The NMR analysis confirmed the presence of 6-gingerol in the extract. The coating of 6-Gingerol on aligners was confirmed by FTIR spectroscopy. The wear resistance of aligners coated for 5 minutes, 10 minutes, and 15 minutes was 1.8 ± 0.09 N, 2.3 ± 0.021 N, and 3.06 ± 0.17 N, respectively, and the difference was statistically significant (p<0.05). The aligner coated for 15 minutes exhibited the widest zone of inhibition of up to 2.38 ± 0.44 mm against *Streptococcus* *mutans*, and the difference was statistically significant (p<0.05).​​​​​​* *No antibacterial effect was found against *E. Coli*.

Conclusion

A novel coating material with 6-gingerol extract incorporated in chitosan biopolymer was prepared and characterized, followed by coating on thermoformed aligners. The coating showed antibacterial activity against *Streptococcus mutans, *and both the antimicrobial activity and wear resistance increased with coating duration.

## Introduction

Aligner therapy, or clear aligner treatment (CAT), refers to the application of clear thermoformed plastics that cover many or all of the teeth’s surfaces and deliver orthodontic forces for the correction of various malocclusions by virtue of attachments on selected teeth. The patient has to remove them while eating and while cleaning their teeth, thus contributing to better oral hygiene and a lower tendency to develop caries [1-3]. Systematic reviews by Cardoso PC et al. and Pereira D. found that aligner therapy is associated with lower pain levels and discomfort as compared to fixed appliance therapy [4,5]. It is because of these advantages over conventional fixed appliances that aligners are trending towards becoming the first choice of orthodontic treatment for patients with aesthetic concerns and compromised periodontal health [6,7].

However, aligners also allow plaque biofilm formation owing to the microcracks and corrugations on their surface and increased surface roughness after thermoforming, leading to bacterial adhesion [8]. The growth of oral microbial pathogens like *Tannerella forsythia*, *Prevotella intermedia*, *Fusobacterium nucleatum*, and *Porphyromonas gingivalis* has been noted as early as two months after the start of clear aligner therapy [9]. A study by Yan et al. investigated microbiological changes on the aligner surface after short-term use. They found that the use of aligners for 12 hours or more creates an adverse trend for an acid-induced demineralization environment due to the abundance of *Streptococcus* and *Lactobacillus sp.* [10]. Also, in a study by Low et al., irregular surfaces of aligners provided protected areas in which bacteria were sheltered from shearing or dislodging forces that are common in the oral cavity. Initial bacterial colonization was found as early as 6-12 hours of appliance wear [11]. Studies have investigated the effect of various cleaning and disinfection protocols on reducing this microbial accumulation [8]. Recent literature on aligner material has focused on functionalizing the aligner surface to prevent plaque from depositing on the aligner. In a study by Xie et al., 4,6-diamino-2-pyrimidinethiol-modified gold (AuDAPT) nanoparticle coating on aligners was reported to inhibit the growth of *P. gingivalis* and *S. mutans* [12]. Worreth et al. applied Cinnamaldehyde (trans-cinnamaldehyde 99%, Sigma-Aldrich) as an antimicrobial agent in a thermofoil aligner coating and reported a decrease in the growth rate of *Staphylococcus epidermidis*, *Streptococcus mutans*, and *Streptococcus mitis* [13]. There is a need for a coating that can be prepared using easily obtainable materials and simple methods without employing a complicated synthesis process.

Ginger is a major part of the diet in most Asian countries and is commonly available. Ginger root (Zingiber officinale Roscoe) has antioxidant, anti-carcinogenic, and anti-inflammatory properties. These have been attributed to its main secondary metabolites, i.e., gingerols [14,15]. Among the gingerols, 6-gingerol (6G) is the most abundant secondary metabolite present in ginger oleoresin [16]. 6-Gingerol in dentistry has been used previously to prevent salivary acinar atrophy and has been shown to have a protective effect [17]. 6-Gingerol was also included as the main active agent in a mouthwash, where it was reported to have anti-inflammatory and anti-microbial activity [18]. Chitosan is made by the deacetylation of chitin, has antifungal and antibacterial mucoadhesion properties, and is non-toxic, biodegradable, and biocompatible. These properties render chitosan useful in dentistry, where it has been proven to improve the compressive strength of the implant coating [19]. 

This study aimed to prepare a 6-Gingerol-based chitosan coating on the surface of thermoformed aligners, characterize it and evaluate its antimicrobial properties and scratch resistance.

## Materials and methods

This present in vitro study was conducted at Saveetha Dental College and involved the below-mentioned steps.

Materials and equipment used for the study

A Duran (Scheu, Germany) thermoformed aligner sheet of thickness 0.5 mm was used for coating. The coupling agents used in this study were Polyethyleneimine (Sigma-Aldrich, Germany), zingerone, and chitosan, which were obtained from SRL Chemicals, India. Carbon-13 Nuclear magnetic resonance (13-c NMR) spectroscopy was done using a 400MHz NMR Spectrometer (Bruker), a nanoindentation test using a mechanical step surface testing platform (Anton Paar Step 700), and a Fourier transform Infrared Spectroscopy (FTIR) test using an Alpha II-Compact Fourier transform Infrared Spectrophotometer (Bruker).

Preparation of aligner samples

**Figure 1:**
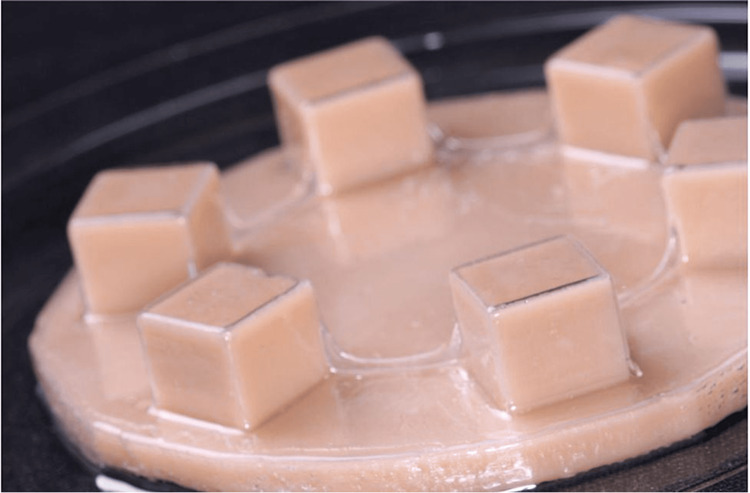
Template for thermoforming the aligner cubes

A template was designed in the graphic design software (Autodesk 3ds Max) to form cubes of 1 cm dimension on a platform of 10 cm diameter. The template was 3-D printed in resin (NextDent 5100), and thermoforming of the aligner sheet material to cubes of a dimension of 1x1cm was done using a thermoforming machine (Biostar, Scheu). Aligner sheet material (Duran 0.5 mm sheet, Scheu) was thermoformed on the printed blocks, and then aligner sheet cubes were isolated from the template (Figure 1).

Preparation of 6 -gingerol extract and chitosan coating solution

Extraction of 6-Gingerol 

To prepare the extract, 250 mg of Zingerone was dissolved in 10 ml of ethanol and continuously stirred overnight using a magnetic stirrer operating at 700 rpm. 

Preparation of Chitosan Solution

0.5 grams of low molecular weight chitosan was added to 25 ml of a 2% acetic acid solution and left to be mixed overnight using the magnetic stirrer at 700 rpm.

Coating of Samples in Control Group

Five thermoformed aligner material cube samples were subjected to UV irradiation in a UV chamber for 20 minutes for surface activation. Following this, they were dip-coated with a crosslinking agent, i.e., polyethyleneimine, for 10 minutes. The coated cubes were kept in a hot air oven at 50°C for one hour. Last, these were dip-coated in the chitosan solution for 15 minutes and baked at 50°C in a hot air oven for an hour.

Preparation of Coating Solution

**Figure 2:**
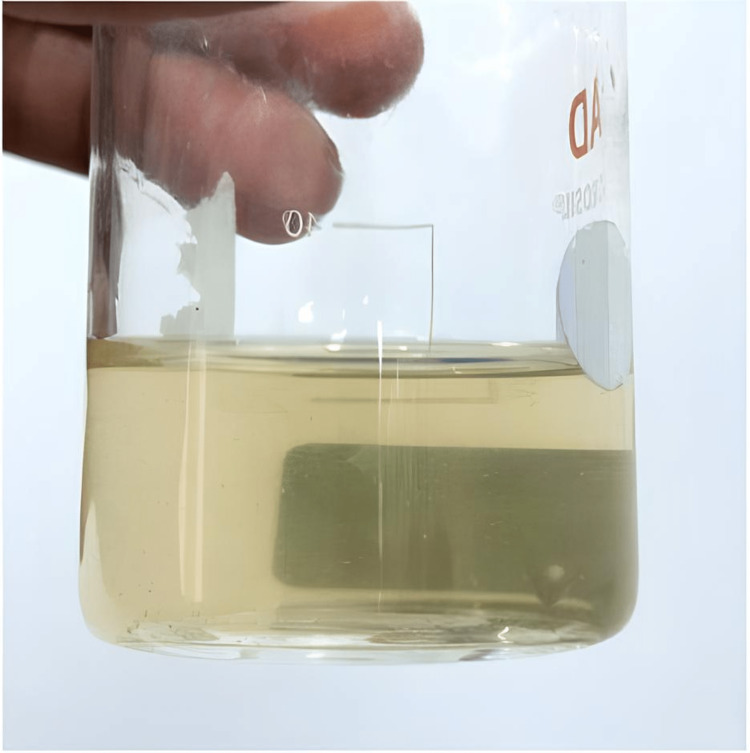
6-Gingerol-Chitosan coating solution

6-Gingerol-Chitosan solution was prepared following the methodology of Mahady et al. [20]. 250 mg of Gingerol extract was mixed in 10 ml of chitosan solution to make a coating solution (Figure 2), the minimum inhibitory concentration (MIC) of which would be 25 micrograms per milliliter.

Coating of the Aligner Samples

**Figure 3:**
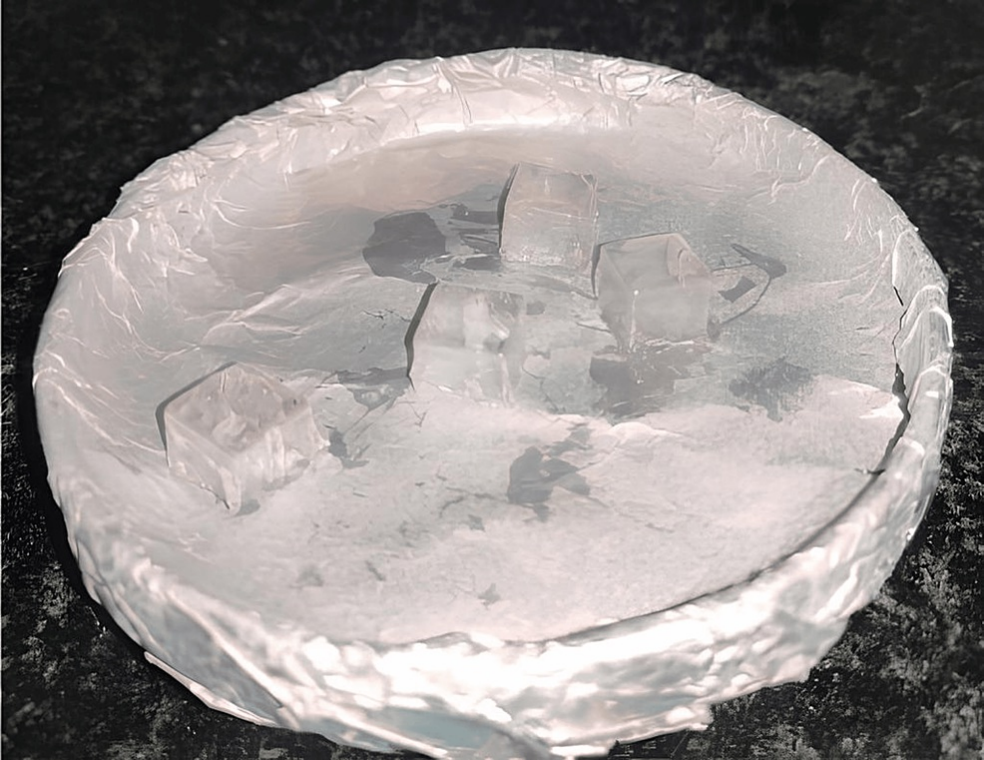
Aligner cubes coated with polyethyleneimine

15 thermoformed aligner material cube samples were subjected to UV irradiation in a UV chamber for 20 minutes for surface activation. Following this, they were dip-coated with a crosslinking agent, i.e., polyethyleneimine, for 10 minutes. The coated cubes were kept in a hot air oven at 50°C for one hour. Figure 3 shows the aligner cubes after baking the polyethylene layer.

**Figure 4:**
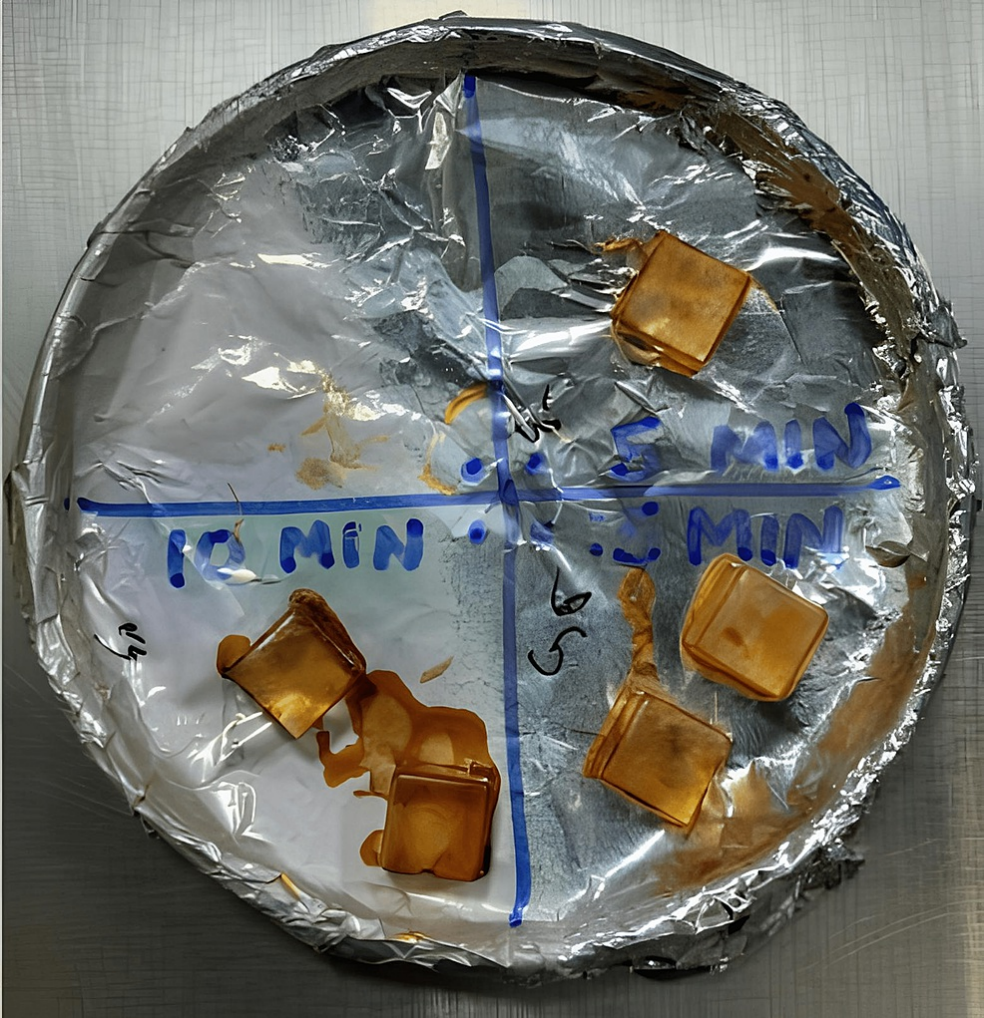
The coated aligner cubes

Last, these were dip coated in the coating solution for 5 minutes, 10 minutes, and 15 minutes and baked at 50°C in a hot air oven for an hour. The aligner cubes were labeled as G5, G10, and G15, respectively (Figure 4). The control was dip-coated only in the chitosan solution without 6-gingerol extract.

Characterization of 6-gingerol

Chemical characterization studies of 6-Gingerol were done using 13C-NMR Spectroscopy (400 MHz, Bruker). To confirm crosslinking between the coating and aligner material, a FTIR test was carried out using a Bruker Alpha II, a Compact Fourier Transform Infrared Spectrophotometer with platinum attenuated total reflectance (ATR).

Nuclear Magnetic Resonance Analysis

NMR samples were prepared by dissolving 30-40 mg of the synthesized 6-Gingerol in 500 μL of CDCl3 as an internal standard. NMR spectra were recorded at 25°C on a Bruker Avance 400 MHz FT-NMR spectrometer, and the obtained spectra were processed using Mnova NMR 14.3.3 software (Mestrelab Research, S.L., Spain).

Fourier Transform Infrared Spectroscopy

The chemical groups of the coated aligners were investigated by attenuated total reflectance - fourier transform infrared spectroscopy (ATR-FTIR) (Bruker, Germany; Model: Alpha II-Compact) in the spectral region of 4000 to 550 cm-1 wavelength with 4 cm−1 resolution at total scans of 32.

Disc Diffusion Test

A disc diffusion test was carried out to evaluate the antimicrobial effect of the coating against Streptococcus Mutans and E. coli. 100 microliters of test organisms were spread onto nutrient agar plates under sterile conditions. 5 mm-diameter samples dipped in the coating solution for 5, 10, and 15 minutes, as well as a control with chitosan coating, were sterilized by UV radiation for 30 minutes. The sterilized discs were kept on the surface of the inoculated agar plates and incubated at 37° C for 24 hours. After 24 hours, the inhibition zone formed around the discs was visually observed, measured, and recorded by a digital camera. The zone of inhibition was repeated three times for each sample.

Determination of Scratch Resistance of the Coating

A nanoindentation test was performed using a mechanical step surface testing platform (Anton Paar Step 700) with a Rockwell type indenter to determine the scratch resistance of the coated aligner material. Force in the range 0-5 N was applied to the sample at a rate of 10 mN min−1, maintained at 5 mN for 30 s, and then unloaded in a process that reversed the loading process. A post-scratch atomic force microscopy analysis was carried out to qualitatively assess the scratch resistance of the coating.

## Results

NMR analysis

**Figure 5:**
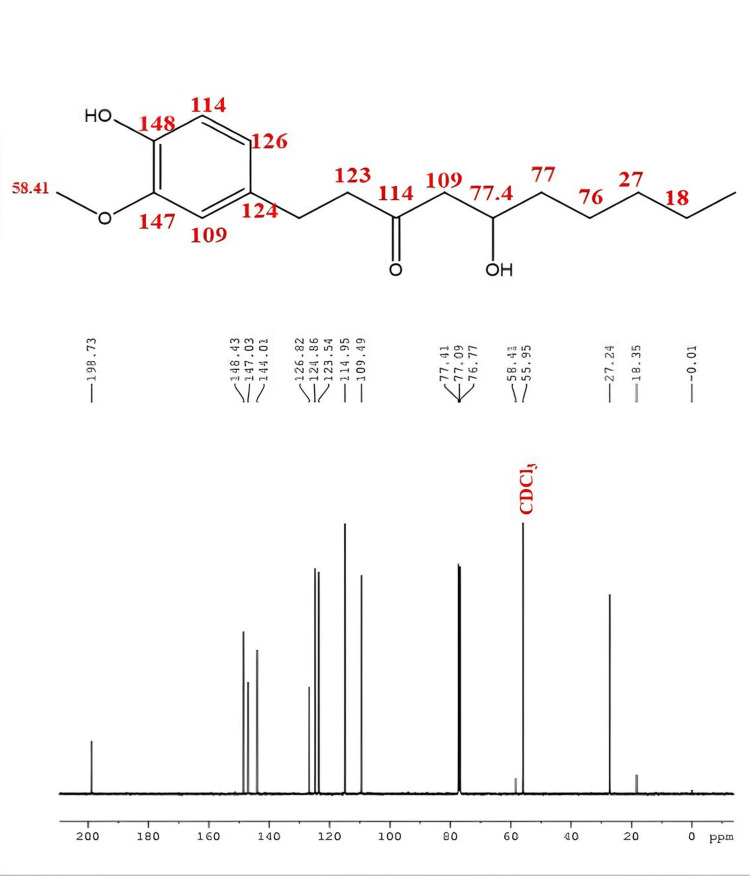
13C-NMR spectrum of 6-gingerol

13C-NMR studies of 6-Gingerol confirmed the presence of 17 carbon skeleton structures with -OH functional groups as a branched chain (Figure 5). 13C-NMR spectral data were tabulated (Table 1), and the structure of 6-Gingerol was characterized using 13C-NMR spectral results by comparing with earlier published literature through the Carbon NMR Database (www.nmrdata.com).

**Table 1:** 13C-NMR spectral values of 6-gingerol

δ_C_	^13^C
18.35	C-10, q
27.24	C-9, (C-8), t
58.47	C-7, t
55.95	C-6, t
77.77	OCH_3_, q
77.41	C-4, d
109.49	C-3, s
114.95	C-2, s
123.54	C-1, s
124.86	C-1’, s
126.82	C-2’, s
144.01	C-3’, s
147.03	C-4’, s
148.43	C-5’, s
198.73	C-6’, s

FTIR test

**Figure 6:**
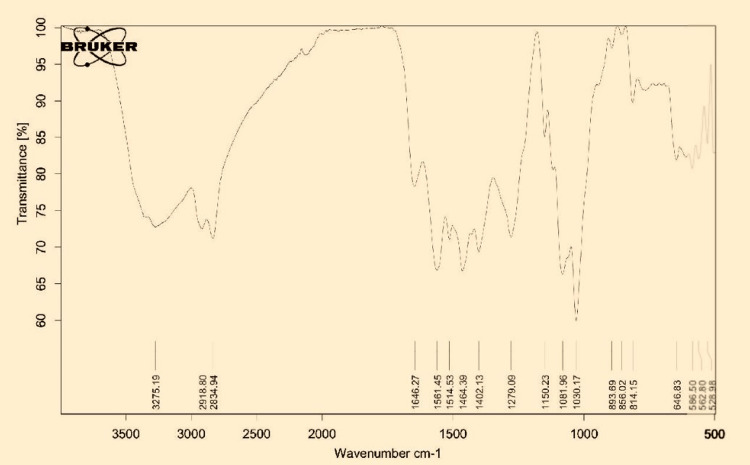
FTIR spectroscopy of 6-gingerol

The FTIR test confirmed cross-linking of the coating and the aligner material, as well as the presence of chitosan molecules within the polymer structure of the aligner material. The characteristic spectral peaks of thermoformed aligners were observed at 1708 cm−1, 1668 cm−1 (stretching mode of the C=O groups, free and H-bonded, respectively); 1527 cm−1 (N-C=O moiety vibrations); 1456 and 1412 cm−1 (bending modes of C-H bonds); 1242 cm−1 (stretching modes of C-N bonds); 1094 cm−1 and 1012 cm−1 (stretching modes of C-O-C bonds); and 720 cm−1 (bending modes of C-H bonds in aromatic rings) (Figure 6). Aligners are primarily made of high-density polyurethanes and polyesters. FTIR spectra are in clear accordance with the high-density polyurethanes and polyesters. Due to the coating of gingerol extract, the above-mentioned characteristic peaks of thermoformed aligners were altered, and a new peak was observed at 3302 cm-1, which corresponds to the alcohol and phenol (O-H) groups of gingerol. It confirms that gingerol was successfully coated on the thermoformed aligners.

Antibacterial effect of 6-gingerol

**Figure 7:**
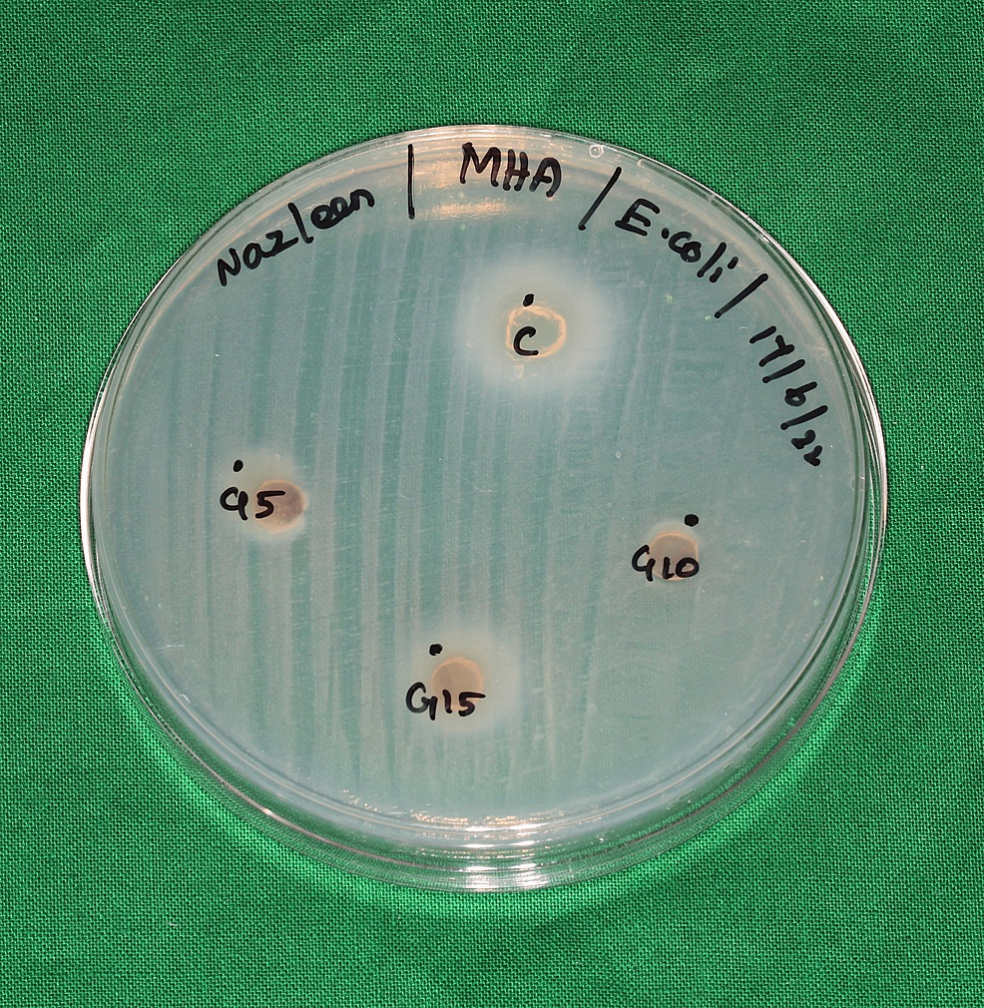
A nutrient agar plate inoculated with E. Coli

**Figure 8:**
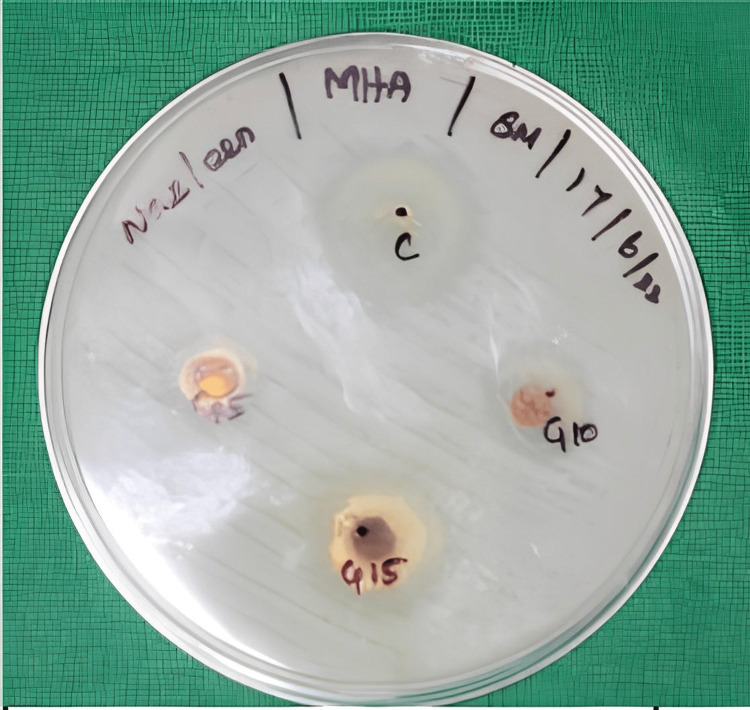
Antibacterial effect of Gingerol-Chitosan biopolymer against S. mutans.

The antibacterial effect of Gingerol-Chitosan biopolymer coating after immersion for 5, 10, and 15 minutes was assessed against *E. coli* and *S. mutans,* and the results were compared with a control that had only chitosan coating. The Gingerol-Chitosan coating doesn’t exhibit any antibacterial effect against E. coli (Figure 7). The zone of inhibition formed against S. mutans in the tested groups is mentioned in Table 2. The measured values were tested for normality using the Shapiro-Wilks test, followed by a one-way ANOVA. A significant difference (p<0.05) was noted, with the highest ZOI formed against S. mutans in the control group, followed by the aligners coated for 15 minutes (Figure 8).

**Table 2:** Comparison of the zone of inhibition formed against SM between the control and the three test groups.

Group	N (number of samples)	Zone of inhibition (mm)	F	P-value
Control	5	4.4 ± 0.54	97.548	0.000
G5	5	0		
G10	5	1.8 ± 0.44		
G15	5	2.8 ±0.44		

Nanoindentation test

**Figure 9:**
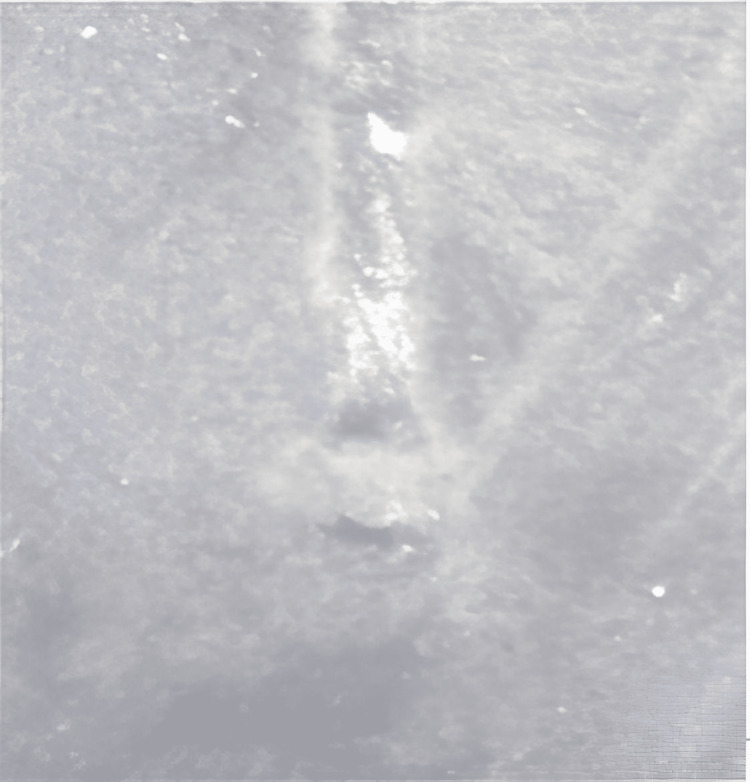
Sample G5 as seen under the scanning electron microscope at 50 X magnification after the scratch test

**Figure 10:**
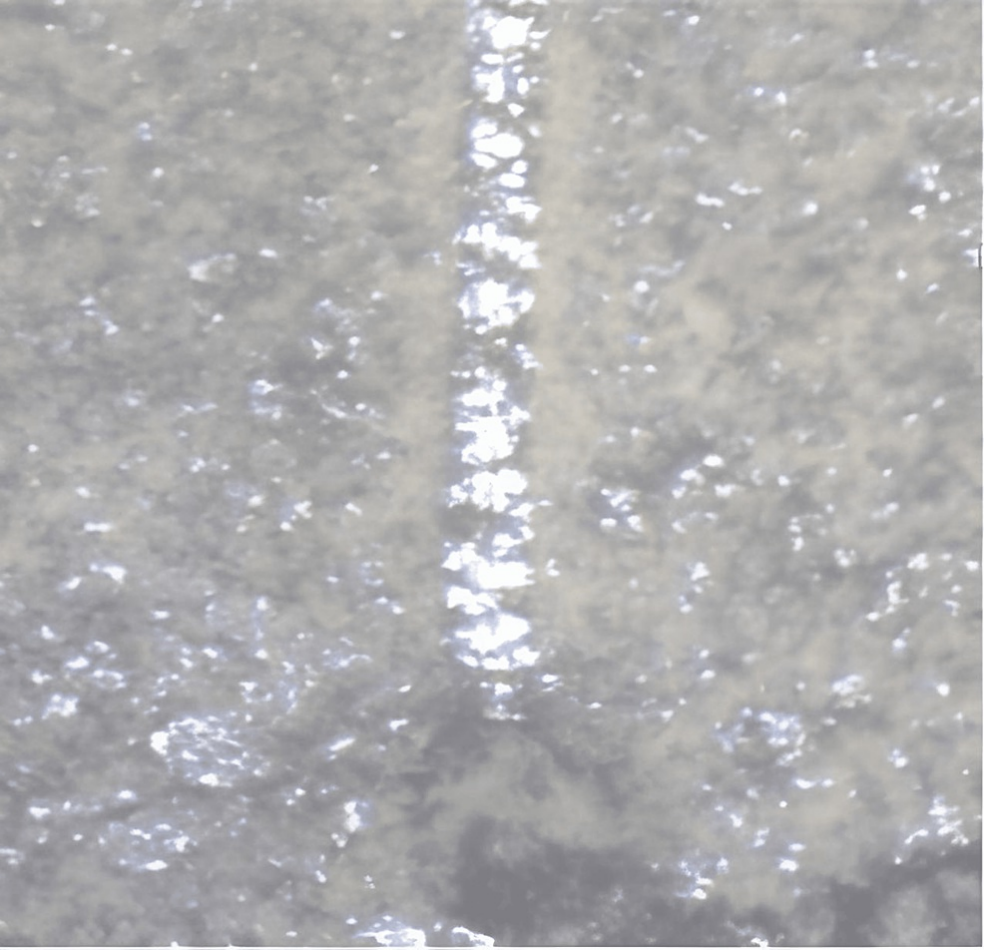
Sample G10 as seen under the scanning electron microscope at 50 X magnification after the scratch test

**Figure 11:**
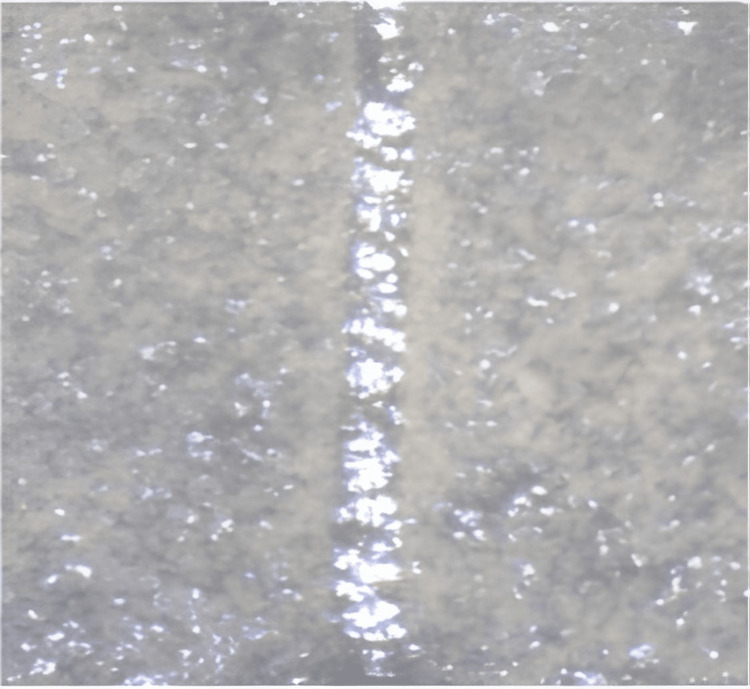
Sample G15 as seen under the scanning electron microscope at 50 X magnification after the scratch test

As shown in Table 3, the force required to indent the surface of the coating increased as the duration of the dip coating increased. The aligners dip-coated for 15 minutes displayed the highest scratch resistance, followed by aligners coated for 10 minutes and then 5 minutes. The dip coating of the aligner material for a longer period thus increases the thickness of the coating (Figures 9-11). The measured values were tested for normality using the Shapiro-Wilks test, followed by a one-way ANOVA.

**Table 3:** Comparison of scratch resistance of aligner cubes dip-coated for five, 10 and 15 minutes.

Group	N (number of samples)	Force (N)	F	P-value
G5	5	1.8 ± 0.09	97.048777777777	0.000
G10	5	2.3± 0.21		
G15	5	3.0 ±0.17		

## Discussion

Thermoforming increases the surface roughness of aligner materials, which leads to bacterial adhesion and plaque establishment on the surface [21]. Coating of the aligner materials to lower the risk of plaque deposition has been attempted in the past. To the best of our knowledge, the application of a Gingerol-Chitosan biopolymer coating on dental aligner material has not been previously described. Park et al. performed polysaccharide coating on clear overlay appliances. In their studies, they varied the coating time up to 20 minutes. We adopted this coating protocol for our studies. In addition, we also conducted preliminary studies in our lab. We observed that during the shorter duration, i.e., less than five minutes, the polymer-aligner crosslinking was poor, which was due to a lesser interaction with crosslinking agents. However, dipping the sample for a period longer than 15 minutes did not increase the coating thickness due to the hydrophobic repulsion of the chitosan polymer. Hence, we performed the coating at three delineated time intervals of five minutes, 10 minutes, and 15 minutes [22].

The Gingerol-Chitosan biopolymer conjugate was tested for its antibacterial activity against *Streptococcus mutans* and *E. Coli*. The present study exhibited an antibacterial effect against *S. mutans* only. This antibacterial activity increased with an increase in coating thickness. The anti-bacterial activity of gingerol against *E. coli* has been described previously at an MIC of 20 micrograms per milliliter [23]. In our study, the gingerol-chitosan coating produced had no antibacterial effect at an MIC of 20 micrograms per milliliter.

Zhang et al. devised a coating of gold quaternary ammonium (QA)-modified gold nanoclusters on the surface of aligners by the plasma deposition technique. Biofilm formation of *S. mutans* and *Porphyromonas gingivalis* on the coated aligners was assessed using an EnSpire multimode reader to indicate the biofilm biomass and visualized using fluorescence imaging, since *S. mutans* is implicated in the etiology of dental caries and *P. gingivalis* in the pathogenesis of periodontitis. The coated aligners had negligible cytotoxicity and were effective against both *S. mutans* and *P. gingivalis* [12,24]. Worreth et al. reported the use of cinnamaldehyde (trans-cinnamaldehyde 99%, Sigma-Aldrich) as an antimicrobial agent as a thermofoil aligner coating on a thermoformed aligner and studied its antimicrobial effect using isothermal microcalorimetry. The coating was found to be effective in reducing the growth rate of *S. epidermidis*, *S. mutans*, and *S. mitis*. Although effective, both methods are associated with high costs and low clinical feasibility [14]. The present study explored the antimicrobial activity of a gingerol-chitosan coating, both of which are commercially available and relatively inexpensive. The coating of the aligner was achieved with the simple method of dip coating that required a beaker, a pair of forceps, a petri dish, and a hot air oven, which made the coating of aligners feasible in a clinical setting and produced a comparable anti-microbial effect against *Streptococcus mutans*.

Hasan et al. reported that the growth inhibition of *S. mutans* by ginger extract was noted at a minimum inhibitory concentration of 256 μg ml. They attributed the anti-bacterial effect to a reduction in glucan synthesis, bacterial adhesion, and biofilm formation in vitro, and a reduction of caries development when compared to the control group [25]. The extract was prepared in methanol, whereas in the present study, we used an ethanolic extract of ginger with successful anti-microbial activity against *S. mutans*.

Along with gingerol, chitosan was used as it exhibits potent antiplaque activity against several oral pathogens such as *Porphyronomas gingivalis*, *Prevotella intermedia*, and *Actinobacillus actinomycetemcomitans* [26]. The present study evaluated the antimicrobial properties of the coating against *S. mutans* and *E. coli* only. Chemically, chitosan (CHS) is a polymeric material and is composed of N-acetylglucosamine and glucosamine copolymer units, usually available in low, medium, and high molecular weights. Chitosan also showed positive anti-microbial activity against *Streptococcus mutans* in studies by Chavez de Paz et al. [27], which was similar to the results of the present study. Chitosan has free amino groups that allow it to crosslink with the polymers of aligner material. This crosslinking was confirmed in this study using Fourier Transform Infrared Spectroscopy. Its ability to therefore act as a scaffold to hold gingerol as the active agent, as well as its established biocompatibility, made it an appropriate choice of biopolymer in this study. Gu et al. explored the dip coating of chitosan over an Mg-Ca alloy to slow down its biocorrosion; however, no scratch resistance test was performed [28]. In the present study, the thickness of the coating increased with an increase in coating time, and the scratch resistance improved, as confirmed by a Nano-Indentation test. Incorporation of an antimicrobial coating on the aligner material that is always in contact with the entire tooth surface can be advantageous to prevent enamel demineralization during the treatment, and the present study explored a potent coating agent that can be easily obtained and coated in simple clinical settings.

Limitations

The limitations of the study are the small sample size since only aligner cubes were tested. Only the antimicrobial activity and wear resistance of the coating were assessed. The coating was done on a single brand of thermoformed aligner material; the coating properties may vary on different aligner materials. Thus, applying and testing the coating on a wider range of aligner materials with a larger sample size would allow more generalization of results. Another major limitation of this combination includes the staining effect of gingerol on the aligners, which increased with a longer dip coating time and is attributed to Curcumin, desmethoxycurcumin, and 6-dehydrogingerdione compounds present in mature rhizomes [29]. Considering that the main advantage of aligners is their aesthetics, it is necessary that the coating remain colorless. Therefore, to adapt the gingerol extract for use in an aligner coating, it is imperative to depigment the extract. The enzyme Laccase has shown promise in decolorizing industrial and reactive dyes and can potentially be applied to decolorizing gingerol in future studies [30].

Different methods of coating the extract on the aligner can pave the way for future research. Also, the oral environment is complex, with several microbes coexisting. Quorum sensing and biofilm formation by these organisms contribute to their resistance to antimicrobials. In addition, the action of lysozymes and salivary flow and composition on the coating is subject to variation from individual to individual, and further studies simulating in-vivo conditions would be required to study the antimicrobial effect and stability of the coating intra-orally.

## Conclusions

The study successfully characterized and coated 6-gingerol extract. This extract was then incorporated into a chitosan biopolymer and coated on a thermoformed aligner using the dip coating technique. The confirmation of crosslinking between the aligner material and coating was done with FTIR analysis. The coating showed an antibacterial effect against *S. mutans* at an MIC of 20 micrograms/milliliter. The aligner coating of the samples subjected to longer dip coating (15 minutes) displayed both a higher antibacterial effect and scratch resistance. However, the staining property of the coating is a major limitation, and the coating as it is requiring decolorization to be applied for clinical use. Furthermore, testing of the coating after simulation of in vivo conditions would be necessary to ascertain the antimicrobial effect, plaque resistance, and stability of the coating intra-orally.

## References

[1] Richter AE, Arruda AO, Peters MC, Sohn W (2011). Incidence of caries lesions among patients treated with comprehensive orthodontics. Am J Orthod Dentofacial Orthop.

[2] Sundararaj D, Venkatachalapathy S, Tandon A, Pereira A (2015). Critical evaluation of incidence and prevalence of white spot lesions during fixed orthodontic appliance treatment: A meta-analysis. J Int Soc Prev Community Dent.

[3] Buschang PH, Chastain D, Keylor CL, Crosby D, Julien KC (2019). Incidence of white spot lesions among patients treated with clear aligners and traditional braces. Angle Orthod.

[4] Cardoso PC, Espinosa DG, Mecenas P, Flores-Mir C, Normando D (2020). Pain level between clear aligners and fixed appliances: a systematic review. Prog Orthod.

[5] Pereira D, Machado V, Botelho J, Proença L, Mendes JJ, Delgado AS (2020). Comparison of pain perception between clear aligners and fixed appliances: a systematic review and meta-analysis. Appl. Sci.

[6] Rossini G, Parrini S, Castroflorio T, Deregibus A, Debernardi CL (2015). Periodontal health during clear aligners treatment: a systematic review. Eur J Orthod.

[7] Alansari RA, Faydhi DA, Ashour BS (2019). Adult perceptions of different orthodontic appliances. Patient Prefer Adherence.

[8] Low B, Lee W, Seneviratne CJ, Samaranayake LP, Hägg U (2011). Ultrastructure and morphology of biofilms on thermoplastic orthodontic appliances in 'fast' and 'slow' plaque formers. Eur J Orthod.

[9] Sfondrini MF, Butera A, Di Michele P (2021). Microbiological changes during orthodontic aligner therapy: a prospective clinical trial. NATO Adv Sci Inst Ser E Appl Sci.

[10] Yan D, Liu Y, Che X, Mi S, Jiao Y, Guo L, Li S (2021). Changes in the microbiome of the inner surface of clear aligners after different usage periods. Curr Microbiol.

[11] Charavet C, Gourdain Z, Graveline L, Lupi L (2022). Cleaning and disinfection protocols for clear orthodontic aligners: a systematic review. Healthcare (Basel).

[12] Xie Y, Zhang M, Zhang W, Liu X, Zheng W, Jiang X (2020). Gold nanoclusters-coated orthodontic devices can inhibit the formation of Streptococcus mutans biofilm. ACS Biomater Sci Eng.

[13] Worreth S, Bieger V, Rohr N, Astasov-Frauenhoffer M, Töpper T, Osmani B, Braissant O (2022). Cinnamaldehyde as antimicrobial in cellulose-based dental appliances. J Appl Microbiol.

[14] Masuda Y, Kikuzaki H, Hisamoto M, Nakatani N (2004). Antioxidant properties of gingerol related compounds from ginger. Biofactors.

[15] Sanwal SK, Rai N, Jagdish S, Buragohain S. (2010). Antioxidant phytochemicals and gingerol content in diploid and tetraploid clones of ginger (Zingiber officinale Roscoe). Sci Hortic.

[16] Da Silva JA, Sampaio PA, Dulcey LJL, Cominetti MR, Rabello MM, Rolim LA. (2021). Preparation and characterization of [6]-gingerol/β-cyclodextrin inclusion complexes. J. Drug Deliv. Sci. Technol.

[17] Yokoyama M, Haruki M, Fukushima M (2012). Effects of [6]-gingerol on dedifferentiation of salivary acinar cells. Inter Jr Or Med Sci.

[18] Bauer Faria TR, Furletti-Goes VF, Franzini CM, de Aro AA, de Andrade TA, Sartoratto A, de Menezes CC (2021). Anti-inflammatory and antimicrobial effects of Zingiber officinale mouthwash on patients with fixed orthodontic appliances. Am J Orthod Dentofacial Orthop.

[19] Sharifianjazi F, Khaksar S, Esmaeilkhanian A (2022). Advancements in fabrication and application of chitosan composites in implants and dentistry: a review. Biomolecules.

[20] Mahady GB, Pendland SL, Yun GS, Lu ZZ, Stoia A. (2003). Ginger (Zingiber officinale Roscoe) and the gingerols inhibit the growth of Cag A+ strains of Helicobacter pylori. Anticancer Res.

[21] Suter F, Zinelis S, Patcas R, Schätzle M, Eliades G, Eliades T (2020). Roughness and wettability of aligner materials. J Orthod.

[22] Park S, Kim HH, Yang SB, Moon JH, Ahn HW, Hong J (2018). A polysaccharide-based antibacterial coating with improved durability for clear overlay appliances. ACS Appl Mater Interfaces.

[23] Wang X, Shen Y, Thakur K, Han J, Zhang JG, Hu F, Wei ZJ (2020). Antibacterial activity and mechanism of ginger essential oil against Escherichia coli and Staphylococcus aureus. Molecules.

[24] Zhang M, Liu X, Xie Y, Zhang Q, Zhang W, Jiang X, Lin J (2020). Biological safe gold nanoparticle-modified dental aligner prevents the Porphyromonas gingivalis biofilm formation. ACS Omega.

[25] Hasan S, Danishuddin M, Khan AU (2015). Inhibitory effect of zingiber officinale towards Streptococcus mutans virulence and caries development: in vitro and in vivo studies. BMC Microbiol.

[26] Ahmed S, Ahmad M, Ikram S (2014). Chitosan: a natural antimicrobial agent-a review. J Appl. Chem.

[27] Chávez de Paz LE, Resin A, Howard KA, Sutherland DS, Wejse PL (2011). Antimicrobial effect of chitosan nanoparticles on streptococcus mutans biofilms. Appl Environ Microbiol.

[28] Gu XN, Zheng YF, Lan QX, Cheng Y, Zhang ZX, Xi TF, Zhang DY (2009). Surface modification of an Mg-1Ca alloy to slow down its biocorrosion by chitosan. Biomed Mater.

[29] Iijima Y, Joh A (2014). Pigment composition responsible for the pale yellow color of ginger (Zingiber officinale) rhizomes. Food Sci. Technol. Re.

[30] Rodríguez E, Pickard MA, Vazquez-Duhalt R (1999). Industrial dye decolorization by laccases from ligninolytic fungi. Curr Microbiol.

